# An Ethyl Acetate Fraction of *Moringa oleifera* Lam. Inhibits Human Macrophage Cytokine Production Induced by Cigarette Smoke

**DOI:** 10.3390/nu6020697

**Published:** 2014-02-18

**Authors:** Nateelak Kooltheat, Rungnapa Pankla Sranujit, Pilaipark Chumark, Pachuen Potup, Nongnit Laytragoon-Lewin, Kanchana Usuwanthim

**Affiliations:** 1 Faculty of Allied Health Sciences, Naresuan University, Phitsanulok 65000, Thailand; E-Mails: naughty_not@mail.com (N.K.); rungnapapa@nu.ac.th (R.P.S.); pachuenp@yahoo.com (P.P.); 2 Faculty of Science, Naresuan University, Phitsanulok 65000, Thailand; E-Mail: pilaiparkc@nu.ac.th; 3 Department of Radiology, Oncology and Radiation Science, Faculty of Medicine, Uppsala University, Uppsala 75105, Sweden; E-Mail: nongnit.laytragoon-lewin@onkologi.uu.se

**Keywords:** *Moringa oleifera* Lam., cigarette smoke extract, ethyl acetate fraction of *Moringa oleifera* Lam., bacterial lipopolysaccharide, cytokine, human macrophages

## Abstract

*Moringa oleifera* Lam. (MO) has been reported to harbor anti-oxidation and anti-inflammatory activity and useful in the treatment of inflammatory diseases. However, despite these findings there has been little work done on the effects of MO on immune cellular function. Since macrophages, TNF and related cytokines play an important pathophysiologic role in lung damage induced by cigarette smoke, we examined the effects of MO on cigarette smoke extract (CSE)—induced cytokine production by human macrophages. An ethyl acetate fraction of MO (MOEF) was prepared from fresh leaves extract of Moringa and shown to consist of high levels of phenolic and antioxidant activities. Human monocyte derived macrophages (MDM) pre-treated with varying concentrations of MOEF showed decreased production of TNF, IL-6 and IL-8 in response to both LPS and CSE. The decrease was evident at both cytokine protein and mRNA levels. Furthermore, the extract inhibited the expression of *RelA*, a gene implicated in the NF-κB p65 signaling in inflammation. The findings highlight the ability of MOEF to inhibit cytokines (IL-8) which promote the infiltration of neutrophils into the lungs and others (TNF, IL-6) which mediate tissue disease and damage.

## 1. Introduction

Phytochemicals found in medicinal plants have antioxidant activities [1], and are considered useful supplements for treating oxidative stress [2,3]. Leaves of *Moringa oleifera* Lam. (MO) contain alkaloids, flavonoids, glycosides, phenolics, saponins, steroids, and tannins [4,5], which have therapeutic properties as antioxidants, and these have found their use in patients with inflammatory conditions, cancer, hypertension, and cardiovascular diseases [6,7,8].

Cigarette smoke contains a wide range of harmful chemical substances such as acroleine, nitrosamines, polycyclic aromatic hydrocarbons, and oxygen derived reactive species (ODRS) [9,10]. ODRS produced by cigarette smoke also activates immune cells and epithelial lining cells, leading to oxidative stress and damage of the lung and other organs [11,12]. Alveolar macrophages are the prime immune cells that respond to oxidative stress, following exposure to cigarette smoke [13,14]. A pro-inflammatory response is elicited by ODRS through the activation of signal transduction molecules, leading to the activation of the transcriptional factor NF-κB-dependent gene transcription [15,16]. This induces the production of pro-inflammatory cytokines such as TNF-α, IL-6, and IL-8 [17,18]. These promote a neutrophilic infiltration to the lung, leading to inflammation and progression of pulmonary emphysema, chronic obstructive pulmonary disease, and potentially to lung cancer [19,20,21].

Thus MO may provide health benefits to cigarette smokers. It was therefore of interest to see if the MO leaf could inhibit the production of cytokines induced by cigarette smoke extract (CSE) in human macrophages. Our findings show that the ethyl acetate fraction of MO (MOEF) potently inhibits the ability of CSE to induce TNF, IL-6 and IL-8 production in human macrophages. The effects are due to a pre-transcriptional effect of MOEF on macrophage responses.

## 2. Materials and Methods

### 2.1. Plant Materials

Fresh mature leaves of MO were collected from cultivation field located in Phichit, Thailand. The leaves were kept cold and protected from light during transportation and extraction processes. Voucher specimens were also gathered and deposited at The Forest Herbarium, Department of National Parks, Wildlife and Plant Conservation, Bangkok, Thailand, under the voucher specimen number: BKF-180970.

### 2.2. Extractions and Fractionation of MO Leaves

Five kilograms of MO leaves were extracted according protocol described by Verma *et al.* [22]. The leaves were processed through cold solvent extraction by homogenizing with 25 L of acidified aqueous-methanol solution containing 1% acetic acid and 50% methanol. The extract was then filtered to remove the residue and concentrated by evaporating at 40 °C.

MO leaves crude extract was then repeatedly partitioned with diethyl ether and deionized water to separate non-polar fraction from an aqueous part. Sodium bicarbonate was used to adjust the pH of the aqueous part to 8.5, which resulting in denaturation of protein contents and conversion of phenolic acids in the extract to their water soluble sodium salts, before partitioned with chloroform to separate non-phenolic fraction from the extract. The pH of the aqueous part was then adjusted to 3.5 for changing the phenolic sodium salts back to phenolic acid. Finally, ethyl acetate was used to fully fractionate polyphenol from that aqueous part.

Extract and fractions were subsequently air-dried by evaporation at ambient temperature. Fully dried extract and fractions were then weight and stored in airtight containers at −20 °C prior to an analysis and further uses in biological experiments.

### 2.3. Determination of Total Phenolic Content

Total phenolic content of each extract and fractions were measured using Folin-Ciocalteau method modified from Chang *et al.* [2]. Dry samples were dissolved in 50% methanol to reach a final concentration of 100 mg/mL. Using 96-well plate, 2.5 μL of the samples were diluted with 100 μL of deionized water and 25 μL of Folin-Ciocalteau reagent. After 5 min of incubation at room temperature, 100 μL of 2% sodium carbonate solution was add following incubation for 60 min at 50 °C. The absorbance of the samples was then measured at 750 nm using a microplate reader. Total phenolic content were expressed in mg Pyrogallol equivalent/g of dry extract.

### 2.4. Determination of Free Radical Scavenging Activity

Antioxidant activity of extract and fractions were measured by ABTS radical cation decolorization assay modified from method described by Floegel *et al.* [23]. The equal volume of 7.4 mM ABTS^+^ and 2.6 mM of potassium persulfate were mixed and stand for 12 h in the dark to generate ABTS˙ free radical. By using of 96-well plate, 5 μL of each sample were mixed with 100 μL of 1:30 ABTS˙ in methanol. The reaction was incubated in the dark for 2 h and the absorbance at 734 nm was then measured using microplate reader. Anti-free radical activity was expressed as μM Trolox equivalent/100 mg of dry extract.

### 2.5. Isolation and Differentiation of Human Peripheral Blood Monocytes

Leukocytes-rich blood component or buffy coat was obtained from blood bank of Buddhachinaraj Phitsanulok Hospital, Phitsanulok, Thailand with permission. The use of buffy coat for this project received ethical approval from Naresuan University Ethics Committee.

Human monocytes were isolated by density gradient centrifugation using Lymphoprep™ (Axis-Shield PoC AS, Rodeløkka, Oslo, Norway) [24] following by size sedimentation centrifugation using Percoll (GE Healthcare Bio-Sciences AB, Uppsala, Sweden) [25]. Isolated monocytes were maintained in RPMI-1640 (Biochrom AG, Berlin, Germany) supplemented with 10% FBS (Biochrom AG, Berlin, Germany) and 2 ng/mL of M-CSF (United States Biological, Salem, MA, USA). Cells were incubated at 37 °C in humidified atmosphere with 5% CO_2_ for 2 weeks with media replacement every 2 days. Human monocyte derived macrophages (MDM) obtained by these processes were firmly used in the entire experiments in this study.

### 2.6. Preparation of Cigarette Smoke Extract

Cigarette smoke extract (CSE) was prepared from filtered cigarettes (Marlboro^®^ Original, Philip Morris USA Inc., Pittsburgh, PA, USA). By using Borgwaldt RM 20S smoking machine (Heinr. Borgwaldt GmbH, Hamburg, Germany), cigarette smoke was collected in form of particulate matter on 92 mm Cambridge fiberglass filter. The filter was then extracted with absolute ethanol to obtain a cigarette smoke extract, which was further concentrated using rotary evaporator with no heating. The smoke extract was aliquoted and stored protected from light at −80 °C. Nicotine concentration in CSE was measured by liquid chromatography-mass spectrometry (LC-MS-MS) [26].

### 2.7. Determination of Cellular Cytotoxicity

Neutral red uptake cytotoxicity bioassay modified from method of Repetto *et al.* [27] was used to evaluate a cytotoxicity of nicotine (NIC), CSE, Aspirin (ASA), and MOEF. Cells were plated at the density of 5 × 10^4^ cells/well in 96-well plate and incubated for 24 h before an addition of 2-fold pre-diluted test substances. After incubation for 12 h, cells were washed once with Earle's balanced salt solution (EBSS) and maintain in 100 μL of 50 μg/mL neutral red dye in complete RPMI. Cells were further incubated for 2 h in order to let neutral red dye penetrate and accumulate inside the cells. Media containing neutral red dye were discarded; cells were washed once with EBSS and then solubilized with 50 μL of acid-alcohol solution. The absorbance at 545 nm was then measured using micro plate reader. Median lethal concentration (LC_50_) of substances was calculated by dose response relationships/sigmoidal curve fitting analysis. Ten percent lethal concentration (LC_10_) was selected as an appropriate concentration for cellular experiments.

### 2.8. Induction of an Acute Inflammatory Response

Fully differentiated human MDM were treated with either LPS or CSE to stimulate an acute inflammatory response. Cells were plated in 24-well plate at the density of 2 × 10^5^ cells/well following an incubation period of 24 h. Effective concentration of LPS at 10 ng/mL in complete RPMI was given to the cells along with 33 ng/mL nicotine equivalent of CSE, which was an average concentration of nicotine found in smokers [28,29]. Control condition of LPS stimulation was an untreated control cells while pure nicotine was used as a control for CSE level. Cells were then incubated for 12 h to stimulate an inflammatory response.

### 2.9. Investigation of Anti-Inflammatory Activity

Cells were pre-treated with non-toxic concentration of ASA at 341.38 μg/mL and MOEF at 57.53 μg/mL, which were 10% lethal concentration of the substances. ASA was used as a positive control for an anti-inflammatory effect [30]. After 6 h of incubation in normoxic humidified atmosphere, cell were then treated with either LPS or CSE for an induction of acute inflammatory responses.

### 2.10. Enzyme-Linked Immunosorbent Assay (ELISA)

Cell culture supernatant of each experimental condition was used for an evaluation of the expression level of pro-inflammatory cytokines using sandwich ELISA assay described by manufacturer (BioLegend, Inc., USA). Supernatant samples were incorporated with pre-coated capture antibody of TNF-α, IL-6 and IL-8, followed by a specific binding of detection antibody of each cytokines. Signal amplification of avidin-HRP-conjugated secondary antibody was performed. The assay was completed by adding freshly mixed solution of TMB substrate for developing of blue color of the reactions. Finally, 1 M of sulfuric acid was then added to stop the reactions and turn blue color of the reaction to yellow. The absorbances of the reactions were measure at 450 nm with subtraction at 570 nm using micro plate reader.

### 2.11. Total RNA Extraction

Total RNA from cells in each condition were extracted by guanidinium thiocyanate-phenol-chloroform extraction using TRIzol reagent (Life Technologies Corporation, USA). Cells from each experimental condition were directly lysed in their 24-well culture plate using 200 μL of TRIzol reagent with several times of up-down pipetting. After 5 min of incubation, homogenated samples were then transferred to polypropylene micro-centrifuge tube. For phase separation, 100 μL of chloroform were added to the tubes. After vigorously mixing, samples were separated into aqueous phase and organic phase. The aqueous phase was transferred to new tube and then mixed with 100 μL of isopropanol following a centrifugation at 12,000× *g* for 15 min at 4 °C. Participated RNA pellets were collected, washed once with 500 μL of 75% ethanol and centrifuged at 7500× *g* for 5 min at 4 °C. Finally, RNA was re-suspended with ribonuclease-free water before keeping at −70 °C for further uses and long-term storage. Total RNA of each samples were quantified by measuring an absorbance at 260 nm and 280 nm using NanoDrop ND-1000 spectrophotometer (Thermo Fisher Scientific Inc., Waltham, MA, USA).

### 2.12. Gene Expression Analysis

Expression of pro-inflammatory genes of cells from each conditions were analyzed by One-step qRT-PCR assay using EXPRESS One-Step SYBR^®^ GreenER™ kits (Life Technologies Corporation, Grand Island, NY, USA) following a protocol described by manufacturer. Equally diluted RNA of each samples were converted to their complementary DNA by reverse transcription reaction followed by real-time polymerase chain reaction along with quantitation of pro-inflammatory genes, which specifically amplified by their forward and reverse primer. Genes associated in inflammatory responses used in this study were tumor necrotic factor alpha gene (*TNF*), interleukin-6 gene (*IL6*), and interleukin-8 precursor (*IL8*). V-rel reticuloendotheliosis viral oncogene homolog A (avian), transcript variant 2 gene (*RelA*) was used to observe an upstream regulation of pro-inflammatory genes. Human β actin gene (*ACTB*) was also used in gene expression analysis as a housekeeping gene for qPCR signal normalization. Oligonucleotide sequence of primer pairs specific to those genes were obtained from PrimerBank database [31] as shown in Table 1. Analysis of gene expression was done by normalized gene expression using 2^−∆∆*CT*^ method [32].

### 2.13. Experimental Design and Statistical Analysis

All experiment conditions were set in triplicate for an accurate laboratory results. Three-independent batches of experiment were performed. Laboratory results were statistically analyzed for their significance using GraphPad Prism (GraphPad Software, Inc., La Jolla, CA, USA) with a confidential interval of 99% (*p*-value = 0.01). ANOVA was used for data comparison among experimental conditions. Paired-sample *t*-test was used for comparing between two equal samples as well.

**Table 1:** Oligonucleotide primers used in this study.

Gene	Oligonucleotide Sequence (5′→3′)		T_m_ (°C)	Amplicon Size (bp)
***TNF***	*Homo sapiens* tumor necrosis factor, mRNA			
	F:	CCTCTCTCTAATCAGCCCTCTG	60.8	220
	R:	GAGGACCTGGGAGTAGATGAG	60.2	
***IL6***	*Homo sapiens* interleukin 6 (interferon, β2), mRNA			
	F:	ACTCACCTCTTCAGAACGAATTG	60.2	149
	R:	CCATCTTTGGAAGGTTCAGGTTG	61.3	
***IL8***	*Homo sapiens* interleukin 8 precursor, mRNA			
	F:	TTTTGCCAAGGAGTGCTAAAGA	60.1	194
	R:	AACCCTCTGCACCCAGTTTTC	62.5	
***RelA*** *	*Homo sapiens* v-rel reticuloendotheliosis viral oncogene homolog A (avian), transcript variant 2, mRNA			
	F:	ATGTGGAGATCATTGAGCAGC	60.2	151
	R:	CCTGGTCCTGTGTAGCCATT	61.3	
***ACTB***	β actin; β cytoskeletal actin [*Homo sapiens*]			
	F:	CATGTACGTTGCTATCCAGGC	60.8	250
	R:	CTCCTTAATGTCACGCACGAT	60.2	

* Gene encodes NF-κB p65 protein; F, Forward primer; R, Reverse primer.

## 3. Results

### 3.1. Extraction and Fractionation of MO Leaves

A total of 859.25 g of aqueous-methanol crude extract was processed into 4 fraction types, 62.40 g diethyl ether, 42.20 g chloroform fraction, 6.85 g ethyl acetate fraction, and 667.92 g aqueous residual fraction.

### 3.2. Antioxidant Activity of MO Extracts

**Figure 1:**
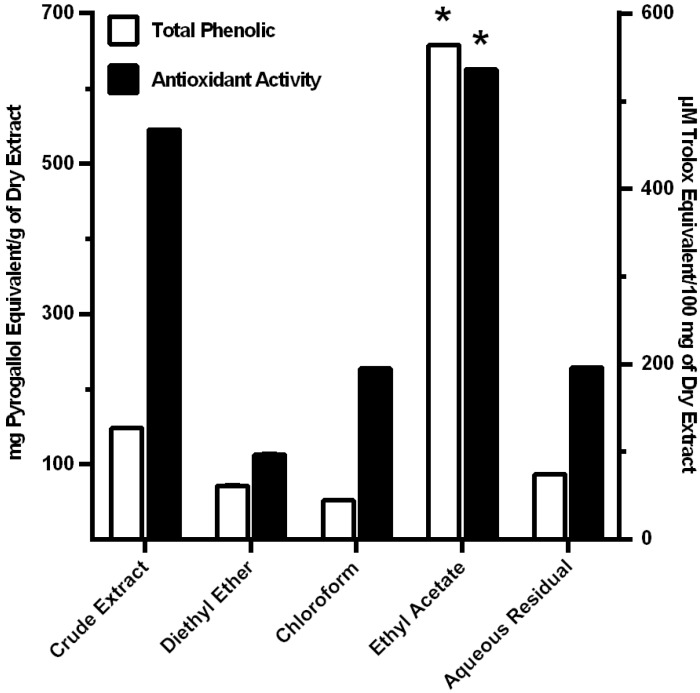
Fractionation of the phenolic and antioxidant activity of *Moringa oleifera*. Total phenolic content was measured by Folin-Ciocalteau method and expressed as mg Pyrogallol equivalen/g of dry extract (□). Antioxidant was measured by ABTS radical cation decolorization assay and expressed as μM Trolox equivalent/100 mg of dry extract (■). Results were analyzed from triplicate data of experiments. * *p* < 0.01 by ANOVA, compared to crude extract and other fractions.

Total phenolic content of crude extract and fractions was determined by the Folin-Ciocalteau method. Total phenolic content of acidified aqueous-methanol crude extract, diethyl ether fraction, chloroform fraction, ethyl acetate fraction, and aqueous residual fraction were 147.79 ± 0.22, 71.35 ± 0.18, 51.96 ± 0.23, 657.45 ± 0.54, and 86.68 ± 0.24 mg Pyrogallol equivalent/g of dry extract, respectively. Antioxidant activity was determined by ABTS radical cation decolorization assay. The anti-free radical activity of acidified aqueous-methanol crude extract, diethyl ether fraction, chloroform fraction, ethyl acetate fraction, and aqueous residual fraction, against a cation radical of ABTS is shown in Figure 1. The data demonstrated that the ethyl fraction had the highest amounts of phenolic, compare to the other fractions (Figure 1). However, in terms of the level of oxidant activity per mg of phenolic, the other three fractions showed higher amounts. This suggests that other compounds in the leaf extract contribute to the anti-oxidant activity.

### 3.3. Cellular Cytotoxicity of Fractions

**Figure 2:**
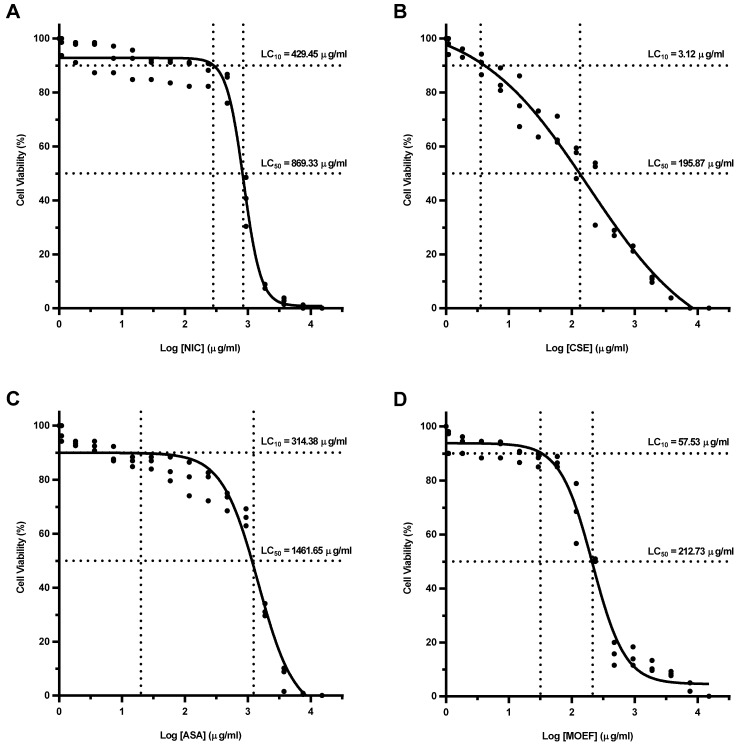
Dose-response curves of each test substances, showing LC_50_ and LC_10_ of NIC; nicotine (**A**); CSE; cigarette smoke extract (**B**); ASA; Aspirin (**C**); and MOEF; ethyl acetate fraction from *Moringa oleifera* extract (**D**). Cells were treated with 2-fold pre-diluted substances for 12 h and tested for their cellular cytotoxicity by neutral red uptake cytotoxicity bioassay. Data of cell viability in triplicate were used to calculate LC_50_ and LC_10_ of each substance by dose-response/sigmoidal curve fitting analysis.

Cytotoxicity of test substances was examined on MDM. The dose-response/sigmoidal curve fitting analysis of percent cell viability were established. LC_50_ of NIC and CSE were 869.33 and 195.87 μg/mL, respectively (Figure 2). A higher LC_50_ was found with ASA at 1461.65 μg/mL. MOEF was effect at a much lower LC_50_, 212.73 μg/mL. LC_10_ of NIC, CSE, ASA, and MOEF were 429.45, 3.12, 341.38, and 57.53 μg/mL, respectively (Figure 2).

### 3.4. Effect on Cytokines Production

**Figure 3:**
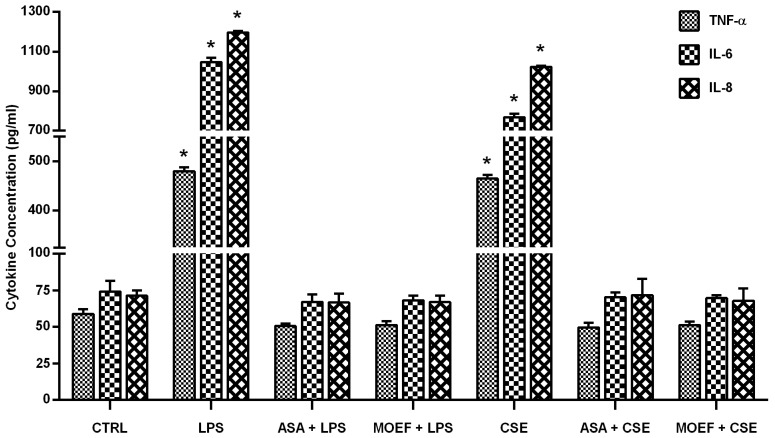
Effects of MOEF on LPS- and CSE-induced tumor necrotic factor alpha gene (TNF), IL-6 and IL-8 production by human MDM. Cells were pre-treated with MOEF, ASA or diluent for 6 h and then stimulated with LPS or CSE. After further 12 h incubation, culture supernatants were harvested to quantify the cytokines. Results are presented as Mean ± SEM of three experiments each conducted with cell from different individual and each in triplicate. Statistical analyses; * *p* < 0.01 by ANOVA, compared to non stimulated control.

MDM were pretreated for 6 h with the extract or diluents and were then stimulated with LPS. After 12 h of incubation cell culture supernatant were collected and cytokines measured by ELISA. Levels of cytokines from each experimental condition are shown in the Figure 3. LPS and CSE caused an approximately 10 fold increase in TNF-α, IL-6 and IL-8 (Figure 3). The data present in Figure 3 demonstrates that both ASA and MOEF reduced the LPS-/CSE-induced cytokine production to basal levels (control). This occurred with respect to TNF-α, IL-6 and IL-8. Of interest was that there was no effect on the basal production of these cytokines, suggesting that MOEF may inhibit the ability of LPS and CSE to induce cell signaling.

### 3.5. Modulation of Inflammatory Gene and Transcription Factor Gene Expression

**Figure 4:**
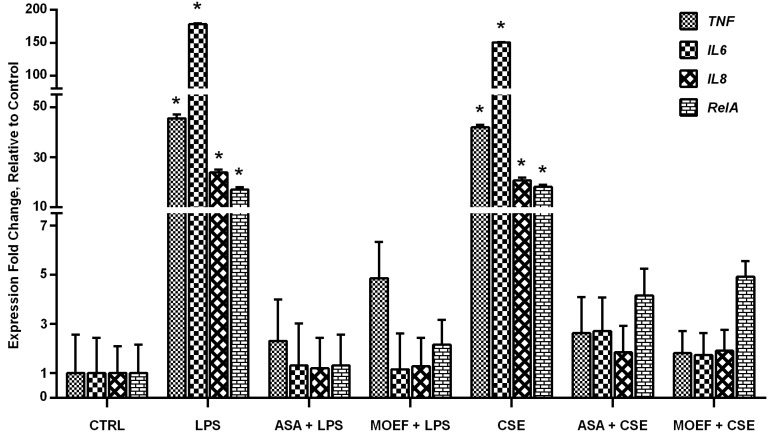
Effects of MOEF on LPS- and CSE-induced *TNF*, *IL-6*, *IL-8* and *RelA* gene expression in human MDM. Cells were pre-treated with MOEF, ASA or diluent for 6 h and then stimulated with LPS or CSE. After a further 12 h incubation culture supernatants were harvested to quantify the cytokines mRNA by one-step qRT-PCR. Results are presented as Mean ± SEM of normalized data of three experiments each conducted with cell from different individual and each in triplicate. Statistical analyses; * *p* < 0.01 by ANOVA, compared to non stimulated control.

Gene expression analysis was accomplished by one-step qRT-PCR, followed by an analysis of normalized gene expression using 2^−∆∆*CT*^ method. Expression of genes from cells in each experimental condition is show in Figure 4. The expression of *TNF* gene from cells stimulated with either LPS or CSE were of the order of 40 fold relative to the non-stimulated control. ASA and MOEF caused almost complete inhibition of expression of the *TNF* gene. ASA and MOEF caused a similar inhibition of the LPS and CSE-induced *RelA*, *IL-6* and *IL-8* gene expression.

## 4. Discussion

Consistent with previous reports [33,34] our data shown that CSE is a strong inducer of TNF, IL-6 and IL-8 in human MDM in culture. This mimics the response of alveolar macrophages following exposure to cigarette smoke [13,14] which leads to the activation of the transcriptional factor NF-κB and release of TNF, IL-1β, IL-6 and IL-8 [17,18], causing leukocyte infiltration and tissue damage in the lung [11,12]. Nutritional approaches in the management of patients with these inflammatory conditions should include medicinal extracts which act on macrophages to prevent the exacerbated inflammatory reaction. Here we demonstrate that MOEF contains anti-oxidant agents which when added to human macrophages caused a marked depression in production of pro-inflammatory cytokines, TNF, IL-6 and IL-8, induced by LPS. These effects were seen in a concentration related manner and at concentrations which had no effect on cell viability.

Another group [35] has reported that an extract of the pod of MO is capable of inhibiting LPS-induced TNF, IL-1β and IL-6 production in the murine macrophage cell line RAW264.7. They also showed that the MO caused a decrease in NF-κB activation. We now not only demonstrate that this is also the case with human macrophages but that MOEF inhibits the response when CSE is used to stimulate macrophages. Thus, this has direct relevance to cigarette smoke induced lung disease.

Macrophages pretreated with MOEF showed significantly decreased production of these cytokines. At non-toxic concentration of MOEF completely inhibited the increase in LPS-induced cytokine production by the macrophages. When macrophages were stimulated with CSE, a similar inhibition of TNF, IL-6 and IL-8 production was caused by MOEF.

Although we have not examined extensively the mechanisms of action by which MOEF bring about the suppression in cytokine production, we found that the extract reduced the expression of an inflammatory gene, *RelA*, implicated in the NF-κB mediated pathogenesis of chronic inflammatory diseases. While we did not examine its effects on NF-κB activation, others have reported that extracts from this plant can inhibit expression and nuclear translocation of the *RelA*/p65 subunit protein of NF-κB [35,36]. Thus, the elevated expression level of *RelA* gene has been found to correlate with its nuclear translocation in the inflammatory response. In our study, we only investigated the expression of *RelA*/p65 subunit protein of NF-κB, which is the most abundant. An inhibitor of NF-κB (IκB) protein, which is the target for anti-inflammatory drug development [37], are principally supposed to inhibit p65-containing complex of NF-κB with noticeably high affinity [38]. Some reports have suggested that MO inhibits kinases upstream of NF-κB, IKB kinase and the MAP kinases [39], which are induced by oxidative stress. This would be consistent with our finding that basal activity was not affected, only that induced by LPS and CSE. Indeed our results extend these mechanisms of the anti-inflammatory properties of MO/MOEF. A reduction in RelA mRNA would give rise to longer term effects on NF-κB and ability to stimulate cytokines in macrophages. Since macrophages and TNF are key participants in the pathogenesis of chronic inflammatory diseases, it is not surprising that MO has been found to be beneficial in treating a wide range of inflammatory conditions [4,6]. Our findings highlight this for cigarette smoke-induced lung disease [14,15].

## 5. Conclusions

The findings reveal aspects of the mechanism of the anti-inflammatory effects of MO which may explain the beneficial effects of this plant in treating chronic inflammatory diseases. This is the first demonstration that a phenolic rich fraction of MO inhibits cytokine production by human macrophages in an *in vitro* model of CSE-induced macrophage TNF, IL-6 and IL-8 production. The data revealed that MOEF depress the expression of *RelA*, a gene important in NF-κB signaling inflammatory reaction. Similar results were found when LPS was used to stimulate these macrophage functions, suggesting an effect on a wider range of macrophage agonists.
